# Supplementary material for: Microbial Keratitis in Nepal: Predicting the Microbial Aetiology from Clinical Features
Source: J Fungi (Basel). 2022 Feb 19;8(2):201. doi: 10.3390/jof8020201 (PMC8879647; doi:10.3390/jof8020201)
Supplement: Supplementary file 1 [file jof-08-00201-s001.zip › jof-1594398-supplementary.pdf]

Supplementary Material

Supplementary Figure S1: Operating characteristic curve showing probability of fungal infection at different scores (mixed infections excluded). (95% CI dashed lines)

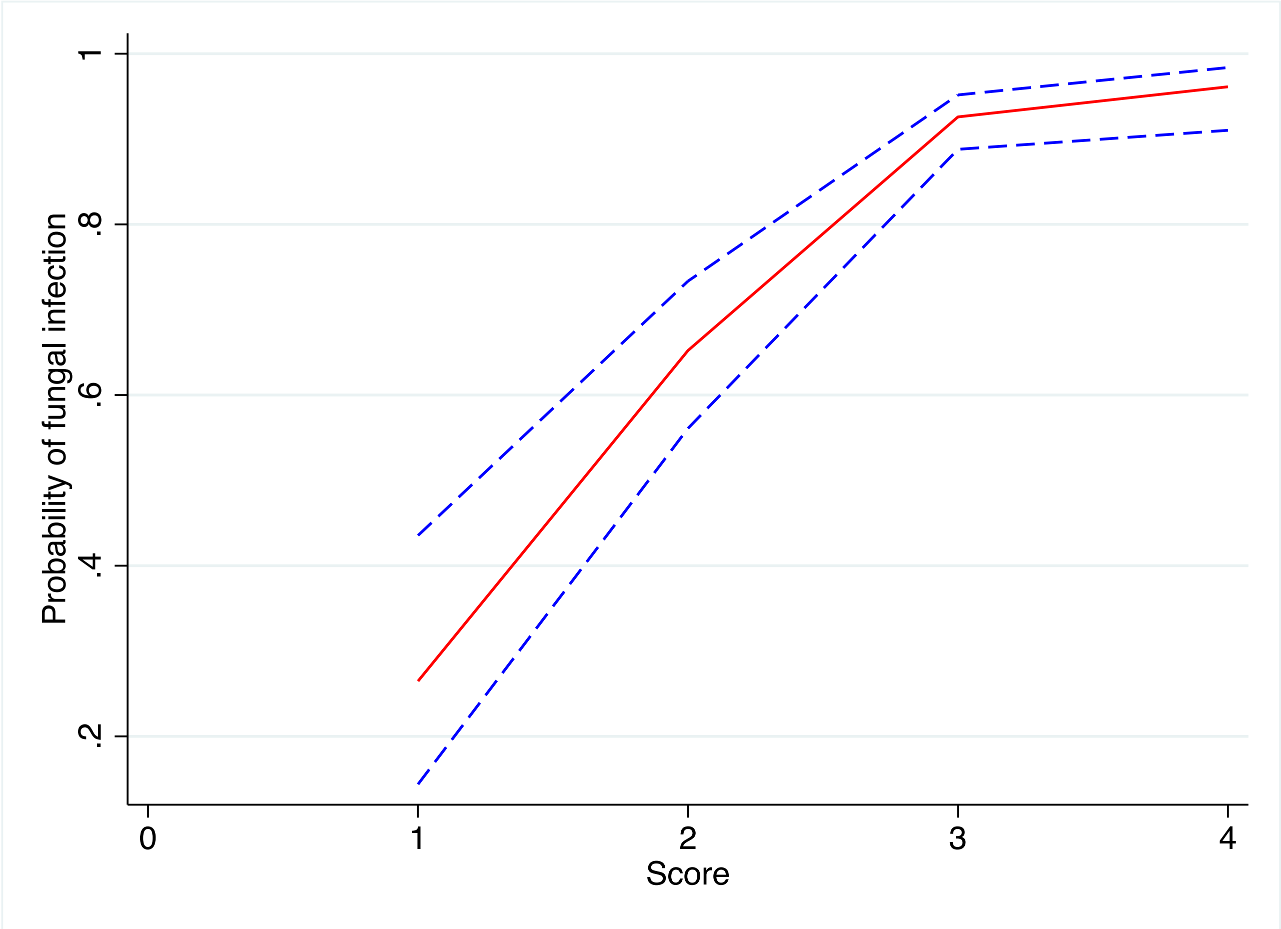

Supplementary Table S1: Reasons for exclusion of potential participants from the study

Reason	n/247	Percent
Did not consent	135	54.7%
Healed or chronic ulcer	55	22.3%
Epithelial defect less than 1mm	40	16.2%
Aged less than 18 years	10	4.0%
Presented out-of-hours	5	2.0%
Clinically viral keratitis	1	0.4%
Mooren’s keratitis	1	0.4%

Supplementary Table S2: Univariable analysis for features associated with fungal keratitis (mixed infections excluded).

	Odds Ratio	p-value	95% CI	Sens	Spec	PPV	NPV
Serrated margins	12.74	<0.001	7.41 21.90	94.2%	44.1%	89.1%	60.8%
Fibrin	1.13	0.784	0.46 2.79	7.3%	93.5%	84.6%	17.1%
Hypopyon	0.63	0.037	0.40 0.97	26.1%	63.9%	76.8%	15.9%
Raised slough	6.55	<0.001	4.20 10.20	82.5%	58.2%	90.0%	42.1%
Satellite lesions	2.29	0.001	1.38 3.81	39.3%	78.0%	89.1%	22.0%
Pigmented colour	1.87	0.313	0.55 6.31	5.0%	97.3%	89.3%	18.3%
Nasolacrimal duct obstruction	0.13	<0.001	0.06 0.28	2.8%	81.8%	41.9%	15.3%
Reduced corneal sensation	0.67	0.153	0.39 1.16	12.9%	81.8%	76.5%	17.1%
Trauma with vegetative object	2.19	0.003	1.31 3.65	34.1%	80.9%	89.1%	21.2%
Previous antibiotics	1.76	0.011	1.14 2.73	75.5%	36.4%	84.4%	24.5%
Delayed presentation > 3 days	1.65	0.066	0.97 2.82	86.9%	20.0%	83.2%	25.0%
Previous steroids	2.45	0.015	1.19 5.03	17.9%	91.8%	90.9%	19.7%

Supplementary Table S3: Multivariable analysis of clinical features occurring in fungal and bacterial keratitis (mixed infections excluded)

	Odds Ratio	p-value	95% CI
Serrated margins	7.32	<0.001	3.95 13.58
Raised slough	4.52	<0.001	2.64 7.72
Nasolacrimal duct obstruction	0.15	<0.001	0.06 0.38
Trauma with vegetative object	2.84	0.006	1.40 5.76

Supplementary Table S4: Screening test indices for each score (mixed infections excluded)

	Sens	Spec	PPV	NPV
Score >0	100%	2.17%	83.6%	100%
Score >1	98%	29.3%	87.4%	75%
Score >2	81.7%	72.8%	93.7%	44.4%
Score >3	27.1%	94.6%	96.1%	20.7%

Supplementary Table S5: Clinical features occurring in dematiaceous, *Fusarium* spp., and *Aspergillus* spp. keratitis, and univariable analysis for features associated with dematiaceous fungal keratitis.

							Indices for detecting dematiaceous fungal keratitis							
	Frequency dematiaceous	(%)	Frequency *Fusarium*	(%)	Frequency *Aspergillus*	(%)	Odds Ratio	p-value	95% CI		Sens	Spec	PPV	NPV
Serrated margins	207/212	98%	57/60	95%	48/54	89%	2.68	0.073	0.91	7.86	97.6%	6.1%	54.9%	68.8%
Fibrin	10/195	5.1%	6/54	11%	3/43	7%	0.52	0.117	0.22	1.18	5.1%	90.5%	40.0%	43.6%
Hypopyon	55/208	26%	20/61	33%	19/54	35%	0.85	0.479	0.55	1.33	26.4%	70.3%	50.5%	45.6%
Raised slough	196/212	92%	53/64	83%	42/54	78%	2.76	0.002	1.47	5.18	92.5%	18.4%	56.5%	68.0%
Satellite lesions	71/196	36%	28/58	48%	24/49	49%	0.65	0.045	0.43	0.99	36.2%	53.3%	48.0%	41.3%
Pigmented colour	23/212	11%	1/64	1.6%	0/54	0%	7.38	0.001	2.18	25.01	10.8%	98.4%	88.5%	49.1%
Nasolacrimal duct obstruction	3/192	1.6%	0/62	0%	0/49	0%	0.53	0.390	0.12	2.25	1.6%	97.1%	37.5%	46.9%
Reduced corneal sensation	17/212	8%	11/64	17%	8/54	15%	0.47	0.019	0.25	0.88	8.0%	84.3%	37.0%	44.4%
Trauma with vegetative object	68/212	32%	16/64	25%	11/54	20%	1.09	0.699	0.71	1.67	32.1%	69.7%	54.8%	47.3%
Previous antibiotics	152/212	73%	50/64	78%	41/54	76%	0.82	0.404	0.52	1.30	73.1%	23.2%	52.2%	43.0%
Delayed presentation > 3 days	193/212	91%	51/64	80%	48/54	89%	1.51	0.202	0.80	2.86	91.0%	13.0%	54.5%	55.8%
Previous steroids	39/212	18%	12/64	19%	13/54	24%	2.45	0.015	1.19	5.03	18.4%	76.8%	47.6%	45.1%

There were no clinical features found to be significantly associated with *Fusarium* or *Aspergillus* keratitis in our sample.

Supplementary Table S6: Multivariable analysis of clinical features occurring in dematiaceous fungal keratitis

	Odds Ratio	p-value	95% CI	
Raised slough	2.78	0.016	1.21	6.38
Satellite lesions present	0.58	0.022	0.37	0.92
Pigmented colour	7.54	0.002	2.11	26.9
Fibrin	0.38	0.039	0.15	0.95
